# Importance of Collateralization in Patients With Large Artery Intracranial Occlusive Disease: Long-Term Longitudinal Assessment of Cerebral Hemodynamic Function

**DOI:** 10.3389/fneur.2018.00226

**Published:** 2018-04-06

**Authors:** Larissa McKetton, Lakshmikumar Venkatraghavan, Julien Poublanc, Olivia Sobczyk, Adrian P. Crawley, Casey Rosen, Frank L. Silver, James Duffin, Joseph A. Fisher, David J. Mikulis

**Affiliations:** ^1^Division of Neuroradiology, Joint Department of Medical Imaging, University Health Network, Toronto, ON, Canada; ^2^Department of Anaesthesia, University Health Network, Toronto, ON, Canada; ^3^Institute of Medical Science, University of Toronto, Toronto, ON, Canada; ^4^Department of Medical Imaging, University of Toronto, Toronto, ON, Canada; ^5^Division of Neurology, Department of Medicine, University of Toronto, Toronto, ON, Canada; ^6^Department of Physiology, University of Toronto, Toronto, ON, Canada

**Keywords:** collateral circulation, intracranial occlusive disease, cerebrovascular reactivity, magnetic resonance angiography, MRI, cortical thickness, neurovascular uncoupling

## Abstract

Patients with large artery intracranial occlusive disease (LAICOD) are at risk for both acute ischemia and chronic hypoperfusion. Collateral circulation plays an important role in prognosis, and imaging plays an essential role in diagnosis, treatment planning, and prognosis of patients with LAICOD. In addition to standard structural imaging, assessment of cerebral hemodynamic function is important to determine the adequacy of collateral supply. Among the currently available methods of assessment of cerebral hemodynamic function, measurement of cerebrovascular reactivity (CVR) using blood oxygen level-dependent (BOLD) MRI and precisely controlled CO_2_ has shown to be a safe, reliable, reproducible, and clinically useful method for long-term assessment of patients. Here, we report a case of long-term follow-up in a 28-year-old Caucasian female presented to the neurology clinic with a history of TIAs and LAICOD of the right middle cerebral artery (MCA). Initial structural MRI showed a right MCA stenosis and a small right coronal radiate lacunar infarct. Her CVR study showed a large area of impaired CVR with a paradoxical decrease in BOLD signal with hypercapnia involving the right MCA territory indicating intracerebral steal. The patient was managed medically with anticoagulant and antiplatelet therapy and was followed-up for over 9 years with both structural and functional imaging. Cortical thickness (CT) measures were longitudinally assessed from a region of interest that was applied to subsequent time points in the cortical region exhibiting steal physiology and in the same region of the contralateral healthy hemisphere. In the long-term follow-up, the patient exhibited improvement in her CVR as demonstrated by the development of collaterals with negligible changes to CT. Management of patients with LAICOD remains challenging since no revascularization strategies have shown efficacy except in patients with moyamoya disease. Management is well defined for acute ischemia where the presence and the adequacy of the collateralization dictate the need for intervention. Long-term assessment in neurovascular uncoupling (i.e., chronic ischemia) may reveal improvements in CVR as the durability of compensatory collaterals improve, even in cases with no intervention. Thus, assessment of cerebrovascular hemodynamics using CVR measurements coupled with time-of-flight MR angiography can be useful in the clinical management of patients with LAICOD.

## Introduction

Hemodynamic stroke is a common presentation of patients with large artery intracranial occlusive disease (LAICOD). Atherosclerosis has been known to be a major factor in the development of LAICOD. Other non-atherosclerotic causes include moyamoya disease, moyamoya syndrome (MMS), fibromuscular dysplasia, dissection, and vasculitis (1, 2). The natural course of LAICOD is quite variable, with the proportion of progression ~9–12% for 6 months (3). Annual stroke risks in symptomatic and asymptomatic patients are 12.5% and 2.8%, respectively (4). The risk of stroke in these patients depends on the adequacy of collateral circulation (5). Collateral circulation is known as a physiologic bypass that attempts to compensate for flow deficits thereby limiting size and growth of an ischemic lesion (6). The status of collateral flow has been shown to influence the rate of arterial recanalization, reperfusion, hemorrhagic transformation, subsequent neurological outcomes, and the overall prognosis of acute ischemic stroke (7–10). Imaging plays an important role in diagnosis, treatment planning, and prognosis of patients with LAICOD (11). In addition to standard structural imaging, assessment of cerebral hemodynamic function is important to determine the adequacy of collateral supply. Here, we report a case of long-term longitudinal follow-up of cerebrovascular hemodynamic function in a 28-year-old female presented with LAICOD of the right middle cerebral artery (MCA).

## Background

For a given degree of angiographic stenosis, some patients have adequate collateral supply, while others lack sufficient collateral flow. Recent advances in brain imaging have improved our ability to identify the latter group. Among the currently available methods of assessment of cerebral hemodynamic function, measurement of cerebrovascular reactivity (CVR) using blood oxygen level-dependent (BOLD) MRI and precisely controlled CO_2_ has shown to be a safe, reliable, reproducible, and clinically useful method for long-term assessment of the patients (12). CVR was measured using precisely controlled CO_2_ as global vasodilatory stimuli and BOLD MRI as a surrogate for measuring cerebral blood flow. A computer controlled gas blender (RespirAct™, Thornhill Research Inc., Toronto, ON, Canada) running a prospective gas targeting algorithm was used for precise targeting of CO_2_, and the details of this technique have been previously published (13). We use this semiquantitative measure of CVR as a measure of the adequacy of collateral circulation and has been shown to be safe, repeatable, and reproducible (14, 15). In patients with LAICOD, the post-stenotic blood vessels often undergo compensatory vasodilation to maintain normal blood flow levels but as a result there is an increasing limitation in the ability of the vascular system to augment flow in the setting of increased flow demand seen with neuronal activation (consumption of vascular reserve). Global vasodilatory stimuli can even lead to paradoxical decreases in CBF in regions with exhaustion of vascular reserve as the blood flow is redistributed from territories that can no longer lower resistance through vasodilation to those that can (16). This phenomenon is known as “intracerebral steal” and is a strong marker for the risk of stroke and a useful parameter to follow when considering revascularizing procedures in symptomatic patients (17).

## Case Presentation

**Figure 1:**
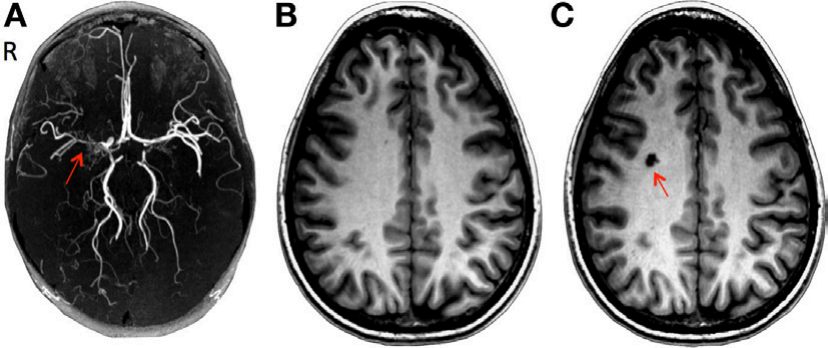
**(A)** Three-dimensional time-of-flight magnetic resonance angiography (MRA): severe stenosis in the right middle cerebral artery (red arrow). **(B)** Brain T1-weighted 3D spoiled gradient echo sequence MRI in June 2007 after initial presentation and **(C)** after the development of a lacunar infarct in the right centrum semiovale (red arrow) during a follow-up in September 2007.

**Figure 2:**
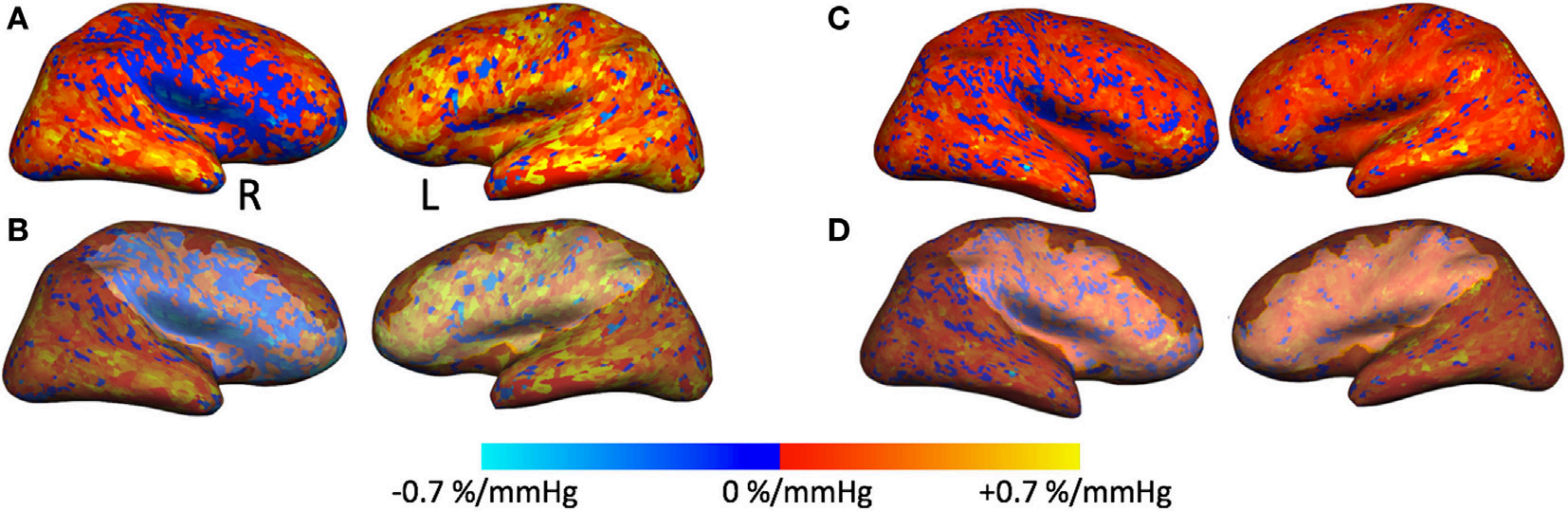
Cerebrovascular reactivity (CVR) maps and regions of interest overlaid on cortical surfaces. **(A,C)** Right and left CVR maps overlaid on inflated cortical surface from the February 2008 time point **(A)** and December 2016 time point **(C)**. **(B)** The same CVR maps as in panel **(A)** that also include the region of interest (ROI) traced around the blue region of steal physiology in the right hemisphere and around the same region with normal CVR in the left hemisphere. These ROI masks were applied to subsequent time points for cortical thickness measurements as exemplified in panel **(D)**.

**Figure 3:**
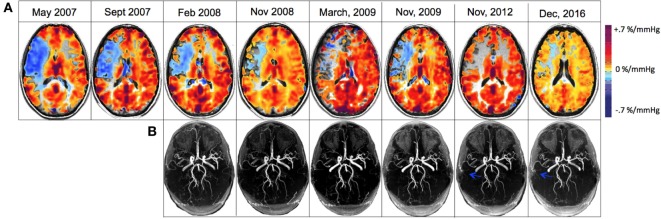
**(A)** Cerebrovascular reactivity maps overlaid on anatomical 3D T1-weighted acquisitions for registration to MNI space for displaying matching coordinates for region analysis. **(B)** 3D time-of-flight magnetic resonance angiography (MRA) of the circle of Willis that were re-registered to one another and slice corrected to include only the identical slices within the brain slab for each acquisition. Blue arrows denote the new collateralized vessels compared with previous scans.

In May 2007, a 28-year-old Caucasian woman presented to the neurology clinic with a history suggestive of transient ischemic attacks (TIAs) affecting the left face and arm. The patient was previously diagnosed with aplastic anemia and paroxysmal nocturnal hemoglobinuria (PNH) in 2004 and started on a monoclonal antibody therapy (eculizumab) for PNH. Her neurological exam including fundoscopy was completely normal. A full work-up for TIA was carried out as per standard practice which included a CT angiography of head and neck and a high resolution 3 T MRI (General Electric Health Care, Milwaukee, WI, USA) using multiplanar sequences with an eight-channel phased array head coil. The MR sequences include a T1-weighted 3D spoiled gradient echo (FAST-SPGR) anatomical sequence, a T2*-weighted echoplanar gradient BOLD sequence for measurement of CVR, a T2-weighted fluid-attenuated inversion recovery (FLAIR), and a time-of-flight MR angiography (MRA). The scan was normal except for a few 1–2 mm non-specific foci of high FLAIR signal within the white matter. The MRA showed a proximal right M1 stenosis (Figure 1A). Her blood work showed marked thrombocytopenia (platelet count of 17,000) and anemia (hemoglobin of 96 g/L). It was theorized that LAICOD in this case might have partly been attributed to PNH (though this is not a known consequence of PNH). As the patient was already on warfarin since 2005, 81 mg aspirin (ASA) was started as prophylaxis for further ischemic events. ASA was discontinued after less than a month due to increased bruising and bleeding. CVR in the index case revealed a large area of impaired CVR with a paradoxical decrease in BOLD signal with hypercapnia involving the right MCA territory, indicating steal physiology (blue color) (Figures 2A and 3A).

In addition, we also measured cortical thickness (CT) on a region of interest (ROI) over the cortical region exhibiting steal physiology on the CVR map using Freesurfer software. An additional ROI was created on the healthy contralateral hemisphere for comparison. For longitudinal assessment (2008–2016), each ROI was copied to subsequent anatomical volumes, and CT measures were derived from these confined ROIs (Figure 2).

During a 4-month follow-up visit, structural MRI revealed the evolution of a 1 cm lacunar infarct in the right centrum semiovale where prior CVR imaging showed intracerebral steal (Figures 1B,C). There was no change in the degree of MCA stenosis, and the CVR map was similar to the previous study. The MRA revealed persistent narrowing of the right MCA with small moyamoya type appearing collateral vessels around the MCA trunk at the base of the brain. Blood work showed a hemoglobin of 82 g/L, WBC count of 3.1 × 10^9^/L. The patient remained on warfarin and was followed yearly for the first 3 years and every 2 years with both structural and functional imaging. Over the next 2 years, the patient had been asymptomatic and her follow up imaging studies (February 2008, November 2008, and March 2009) showed no new lesions in MRI. The CVR maps showed persistent steal; however, there was a trend toward improvement (Figure 3A).

The patient discontinued coumadin in 2015 and was restarted on 81 mg ASA daily. In the recent follow-up (December 2016), the MRI showed no new changes; there continued to be a high-grade stenosis of the proximal right MCA (Figure 3B). The CVR study revealed decreased steal physiology in the right hemisphere compared with the previous study (depicted in blue on CVR maps in Figure 3A). Clinically, the patient had no new symptoms since 2008 except for minor left arm and hand tingling, and from a neurological standpoint, it appeared that the patient had clearly developed adequate collateral supply and hence became a candidate for reduced risk for recurring stroke. She continues to take 81 mg ASA daily.

## Results

The long-term assessment of cerebrovascular hemodynamics in this patient showed an overall improvement in the right MCA CVR by 0.26%/mm Hg PCO_2_ (an increase of 450.4%) after a 5-year follow-up, and by 0.18%/mm Hg PCO_2_ (an increase of 322.9%) after 9-year follow-up compared with the initial visit. Similarly, the left MCA improved by 0.082%/mm Hg PCO_2_ (an increase of 40.1%) after a 5-year follow-up, and by 0.062%/mm Hg PCO_2_ (an increase of 30.9%) after 9-year follow-up compared with the initial visit.

**Figure 4:**
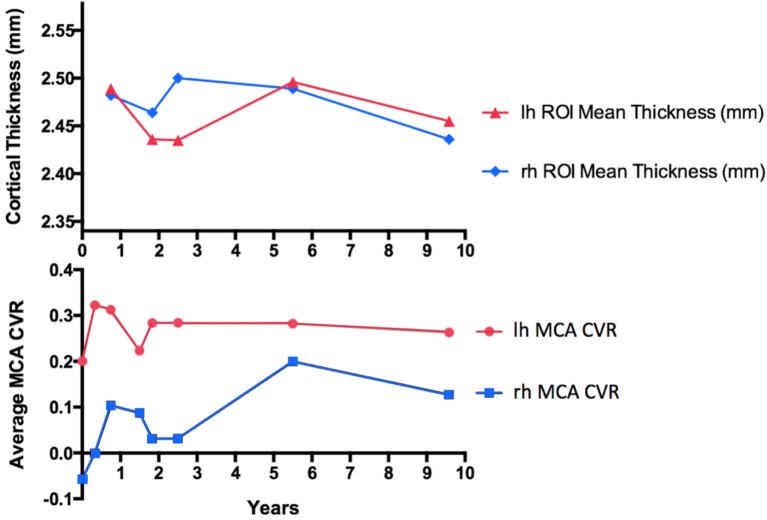
Cortical thickness (CT) measurements compared with mean middle cerebral artery (MCA) territory cerebrovascular reactivity (CVR) change in a patient with large artery intracranial occlusive disease of the right MCA without surgical intervention, followed-up for over a 9-year interval. For CT measurements, only anatomical volumes with the same voxel size and imaging parameters were considered to remove unwanted biases in the longitudinal stream analysis.

Comparing the 4-year follow-up from 2008 to 2012, CT in both the right and left hemispheres increased by 0.007 mm (an increase of 0.28%). After an 8-year follow-up, CT decreased in the right hemisphere by 0.046 mm (a decrease of 1.85%), and in the left hemisphere by 0.034 mm (a decrease of 1.37) (Figure 4). Overall CT remained relatively unchanged in both hemispheres throughout this longitudinal assessment.

## Discussion

To the best of our knowledge, this is the first unique case report of long-term longitudinal assessment cerebrovascular hemodynamic function in a patient presented with LAICOD of the right MCA. Management of LAICOD is challenging since no revascularization strategies have shown efficacy except in patients with MMD or MMS (18). Management is well defined for acute ischemia, and currently there are no treatment options available for chronic cerebral ischemia. Some studies have shown that revascularization can be useful in chronic cerebral ischemia (19). However, in this case, revascularization was not considered since LAICOD had failed to progress since the patient’s PNH was controlled with eculizumab.

Similar to acute ischemia, long-term outcome of chronic ischemia also depends on the adequacy of the collateral supply. Currently, routine assessment of cerebral hemodynamic function is not a standard of care in the follow-up of patients with LAICOD. This may be because of the lack of validated tests to measure CVR. Currently, published CVR studies employ multiple methods of measuring CVR. These methods have not been standardized sufficiently to prevent variability with repeated studies within a subject and less so across subjects (20). We have shown that our technique of measuring CVR using a precisely controlled standardized stimulus is accurate, reliable, reproducible, and can be incorporated into the routine clinical practice (15, 21).

Previous studies have revealed a link between steal physiology leading to neurovascular uncoupling (22) resulting in decreased CT in the ipsilateral hemisphere (23). As such, it is interesting to note that our patient showed improvements in CVR in the MCA territory whereas CT remained relatively unchanged (Figure 4). This has also been observed in MMD following successful revascularization (18). Of note is that the latest time point at year 9 shows what may be the beginning of deterioration in the CVR measures as if the excess flow demand of the collateral vasculature is unsustainable in the long term (Figure 4). Continued follow-up is planned to further assess this possible outcome.

There have been great gains in the management of acute ischemic stroke, but little has been done to detect and manage chronic brain ischemia. Clearly there is a challenge to further elucidate the impact of chronic untreated LAICOD not just on acute but chronic brain injury. Long-term assessment in cerebral hemodynamic function may reveal improvements in CVR as the durability of compensatory collaterals improves, even in cases with no intervention. This longitudinal assessment of a patient with chronic MCA stenosis strengthens a previous cross-sectional study in MMD that links CVR with CT (18). The explanation for this relationship is thought to be reversible injury to the neuropil rather than permanent loss of neurons since the cortex can re-thicken after improvement in CVR with re-establishment of neurovascular coupling (24). It also sheds early light on the efficacy and durability of compensatory collaterals raising questions about the longevity of collateral blood flow compensation. In the future, many questions need to be answered including: (1) What is the relationship between CVR deficits and neuronal function (i.e., cognitive decline)? (2) Are changes in the complexity of the neuropil (i.e., synaptic density and dendritic arborization) the cause of differences in CT or is it related to loss of neurons related (selective neuronal necrosis)? (3) Do collaterals have limited durability and compensatory capacity, and if so, over what time frame? Do they wear out? (4) Is this a mechanism of vascular dementia?

## Conclusion

In summary, the patient presented with TIAs secondary to hemodynamically significant intracranial stenosis. The patient was followed-up over the period of 9 years with both structural and functional imaging to assess CVR and gray matter integrity. Overall, the patient exhibited improved CVR as demonstrated by the development of moyamoya type collaterals with negligible changes to CT. As such, CVR maps coupled with structural imaging (MRA) can be useful in clinical management.

## Ethics Statement

This study conformed to the standards set by the latest revision of the Declaration of Helsinki and was approved by the Research Ethics Board of the University Health Network. Written informed consent was obtained by the patient for the publication of this case report.

## Author Contributions

LM, LV, and DM designed the study. LM analyzed the data and wrote the manuscript. FS cared for the patient. LM, LV, FS, and DM interpreted the data for the work. LM, LV, JP, OS, AC, CS, FS, JD, JF, and DM contributed to the manuscript revision and reviewed and approved the submitted version.

## Conflict of Interest Statement

JF and DM are among the developers of the RespirAct™ for MRI studies at the University Health Network, part of the University of Toronto. Thornhill Research Inc. (TRI) is a for-profit biomedical manufacturing company that was spun off from UHN. It assembles the RespirAct™, on a non-profit basis to enable MRI research at UHN and around the world. JF receives income for work done for TRI and DM holds a minor equity position in TRI. The remaining coauthors declare that the research was conducted in the absence of any commercial or financial relationships that could be construed as a potential conflict of interest.
